# Bilateral Erector Spinae Plane Block for Man o’ War Stings: A Case Report

**DOI:** 10.5811/cpcem.2022.12.58093

**Published:** 2023-02-09

**Authors:** Luke Weber, Michael Shalaby

**Affiliations:** Mount Sinai Medical Center, Department of Emergency Medicine, Miami Beach, Florida

**Keywords:** case report, emergency medicine, regional anesthesia, pain management, Portuguese man o’ war, jellyfish

## Abstract

**Introduction:**

The Portuguese man o’ war, an aquatic invertebrate, is responsible for a large proportion of cnidarian stings worldwide. Cnidaria is a phylum that contains the genus *Physalia*. These injuries result in severe pain and skin irritation, which are often difficult to control. Traditionally, cnidarian stings have been treated by emergency physicians with warm water, vinegar and, in severe cases, opioids. However, no concrete guidelines have been established for pain management in man o’ war stings.

**Case Report:**

Regional anesthesia (RA) is an increasingly used method of pain control in the emergency department. In the case of a 41-year-old female experiencing severe pain from a Portuguese man o’ war sting, RA with an erector spinae plane block (ESPB) provided her with rapid and long-lasting pain relief.

**Conclusion:**

The standard of care has yet to be defined when managing pain from *Physalia physalis* stings. Although this is the first documented use of ESPB for treatment of cnidarian stings, RA should be considered by any emergency physician when treating injuries caused by a Portuguese man o’ war.

## INTRODUCTION

*Physalia physalis*, better known as the Portuguese man o’ war, are frequently found in hot and temperate waters. *Physalia* are responsible for a substantial proportion of cnidarian stings worldwide and are normally quite painful and severe.^1^ Tentacles of *Physalia* can measure from 10–30 meters and envenomate victims via the discharge of nematocysts from these tentacles,^2^ which contain from a few thousand to several billion nematocysts.^1^ After being triggered by mechanical stimuli (ie, skin rubbing or tentacle traction), nematocysts are discharged into the victim within a fraction of a second.^1^ Ensuing nerve irritation and inflammation results in pain, swelling, and itching. *Physalia* stings classically result in linear, crossed, skin wheals.^2^ In more extensive stings, victims can experience skin necrosis or even muscular, gastrointestinal, cardiac, neurological, and allergic symptoms if significant systemic absorption of the toxin occurs.^1^

The erector spinae plane block (ESPB) was originally described by Forero et al (2016) as an effective treatment for thoracic neuropathic pain.^3^ Bilateral ESPB has since been used in a variety of clinical cases, including as a part of multimodal analgesia following thoracic and cardiovascular surgeries, or for chronic low-back pain, vertebral fractures, acute pancreatitis, and rib fractures.^4^–^6^ Risks of the procedure include pneumothorax, nerve injury, and local anesthesia toxicity. However, few complications of ESPB have been published. In our emergency department (ED), for example, we often use the ESPB as part of multimodal analgesia for multiple rib fractures. Opioid avoidance is of particular importance in our ED. Many of our patients are not native to Florida and are opioid-naïve. Opioid-naïve patients requiring an opioid prescription are at greater risk of recurrent opioid use.^7^ Pain control via ESPB allows for avoidance of opioids, clearance of pulmonary secretions, and decreased injury complications.

**Image 1:**
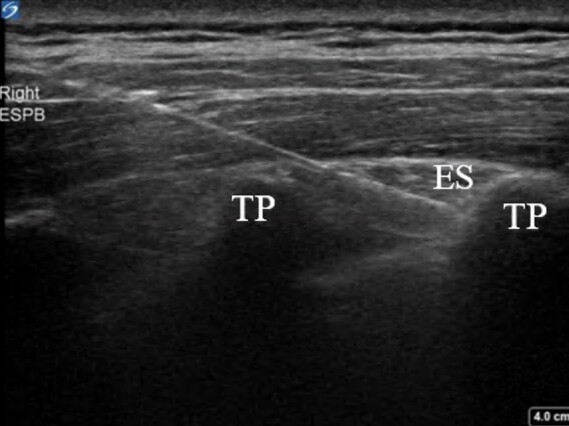
Erector spinae plane block right posterior chest. Ultrasound image of needle placement just prior to anesthesia injection. Transverse processes (TP) and erector spinae muscle (ES) labeled above.

The ESPB is performed by injecting local anesthetic in the posterior chest wall between the erector spinae muscle and transverse vertebral process (Image 1).^8^ With the patient in either orthopneic, prone, or lateral decubitus positioning, the physician stands posteriorly to the patient and chooses the level of spinous process that correlates with the appropriate dermatomal distribution of the patient’s pain.^9^ Under ultrasound guidance, the physician then places the probe longitudinally at the thoracic level to identify the spinous process. Once a proper location is selected, the physician slides the transducer laterally until the transverse process is visualized. While inserting the needle in plane from a cranial to caudad direction, the physician hydro-dissects until the needle contacts the transverse process.^9^ Once the needle is deeper than the erector spinae muscle and at the tip of the transverse process, the physician injects about 20–30 milliliters (mL) of local anesthetic.^8^

Local anesthetic then diffuses anteriorly to the paravertebral and epidural spaces and envelops the ventral and dorsal rami of the corresponding spinal nerves.^8^ The ventral ramus (intercostal nerve) innervates the anterolateral chest wall, while the dorsal ramus innervates the posterior chest wall. Thus, the erector spinae block results in both visceral and somatic analgesia.^7^

## CASE REPORT

**Image 2:**
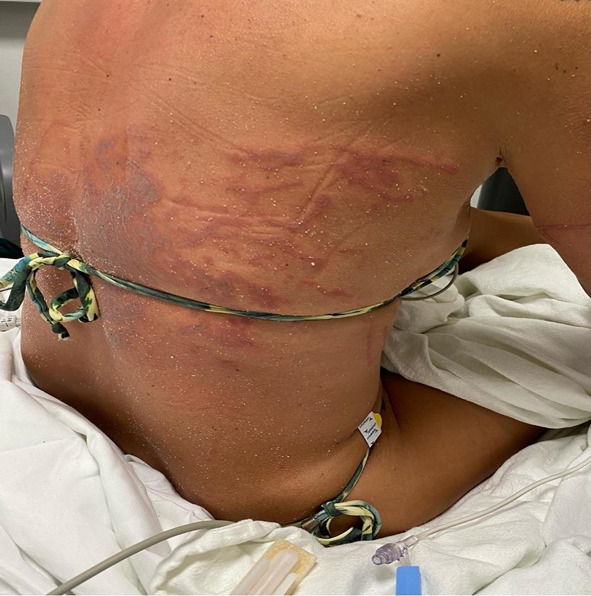
Rashes on posterior chest from Portuguese man-o’-war stings.

A 41-year-old female presented with a chief complaint of painful rash and muscle stiffness following a sting from Portuguese man o’ war one hour prior to arrival, while swimming at a local beach. She related that the tentacles of the jellyfish-like floating invertebrate had attached to her back prior to being dislodged. Her muscle stiffness and pain was so severe that any movement exacerbated her symptoms. Emergency medical services did not provide her with any analgesics. Physical exam was notable for an otherwise healthy-appearing woman in significant distress, with serpiginous, erythematous rashes resulting from man-o’-war stings covering most of her chest and abdomen, also with extensive involvement of her upper arms and thighs (Image 2).

*CPC-EM Capsule*

What do we already know about this clinical entity?

*Identification and treatment of Physalia stings is important in the prevention of life-threatening complications, as well as pain relief for the victim. Initial treatment of Physalia stings should consist of controlling systemic reactions, followed by tentacle removal with warm water irrigation and/or manual removal with tweezers to prevent further nematocyst discharge. As to the best method of pain control, there is much controversy.*

What makes this presentation of disease reportable?

*This patient’s pain was severe, and refractory to opioid administration.*

What is the major learning point?

*Regional anesthesia can provide rapid, thorough, and long-lasting treatment of pain from Physalia stings, and should be considered as part of a multimodal analgesic pathway for patients who present to the emergency department with extensive or severely painful stings.*

How might this improve emergency medicine practice?

*Besides providing patient comfort, regional anesthesia allows the physician to fully inspect wounds and remove additional tentacles without difficulty. As a result, patient outcomes, satisfaction, and disposition times may improve.*

The patient was given 4 milligrams of intravenous morphine without improvement of her pain. Thirty minutes after medication administration, she still rated her pain as 10/10. We then offered an ESPB, to which she consented. Bilateral block administration with 12 mL bupivacaine 0.5% on each side was given, and within another half hour the patient was feeling almost completely better, and after another 15 minutes she was discharged back to her hotel with some mild pain only in her arms and thighs.

Call-back three days later revealed that the patient had experienced 20 hours of complete anesthesia and was feeling much less pain than the initial stings after the anesthetic wore off.

## DISCUSSION

Identification and treatment of *Physalia* stings is important in the prevention of life-threatening complications, as well as pain relief for the victim. Initial treatment of *Physalia* stings should consist of controlling systemic reactions, followed by tentacle removal with warm water irrigation and/or manual removal with tweezers to prevent further nematocyst discharge. As to the best method of pain control, there is much controversy regarding optimal treatment. Remedies such as hot water and seawater application on the affected skin have shown to be helpful for patients with mild pain.^1^,^10^ Vinegar has been traditionally used for pain relief; however, increasing evidence suggests that vinegar may provoke continued venom release from *Physalia* nematocysts.^11^ Other chemicals previously used to treat skin pain, including ethanol and ammonia, may also stimulate nematocyst discharge.^10^ When administered either topically or subcutaneously, lidocaine has been shown to provide superior relief of pain from jellyfish stings when compared to traditional methods and may prevent further nematocyst discharge from tentacles that remain in the skin.^10^

## CONCLUSION

Regional anesthesia can provide rapid, thorough, and long-lasting treatment of pain from *Physalia* stings and should be considered as part of a multimodal analgesic pathway for patients who present to the ED with extensive or severely painful stings. Besides providing patient comfort, RA allows the physician to fully inspect wounds and remove additional tentacles without difficulty. As a result, patient outcomes, satisfaction, and disposition times may improve. Additionally, significant amounts of opioids can be avoided in patients who undergo RA. This case is the first documented use of ESPB in the treatment of *Physalia* stings. Physicians should consider the use of RA more often for similar cases in the ED.
